# Regulation of vascular smooth muscle cell calcification by syndecan-4/FGF-2/PKCα signalling and cross-talk with TGFβ

**DOI:** 10.1093/cvr/cvx178

**Published:** 2017-09-06

**Authors:** Samantha J. Borland, Thomas G. Morris, Shona C. Borland, Mark R. Morgan, Sheila E. Francis, Catherine L.R. Merry, Ann E. Canfield

**Affiliations:** 1 Division of Cardiovascular Sciences, School of Medical Sciences, Faculty of Biology, Medicine and Health, University of Manchester, Manchester Academic Health Science Centre, Manchester, UK;; 2 Stem Cell Glycobiology Group, School of Materials, University of Manchester, Manchester, UK;; 3 Department of Cellular and Molecular Physiology, Institute of Translational Medicine, University of Liverpool, Liverpool, UK;; 4 Department of Infection, Immunity and Cardiovascular Disease, University of Sheffield, Sheffield, UK;; 5 Wolfson Centre for Stem Cells, Tissue Engineering & Modelling, Centre for Biomolecular Sciences, University of Nottingham, Nottingham, UK

**Keywords:** Vascular smooth muscle cells, Vascular calcification, Syndecan-4, Fibroblast growth factor-2, Transforming growth factor-β

## Abstract

**Aims:**

Vascular calcification is a major cause of morbidity and mortality. Fibroblast growth factor-2 (FGF-2) plays an instructive role in osteogenesis and bone development, but its role in vascular calcification was unknown. Therefore, we investigated the involvement of FGF-2 in vascular calcification and determined the mechanism by which it regulates this process.

**Methods and results:**

We demonstrate that FGF-2 expression is increased in vascular smooth muscle cells (VSMCs) induced to deposit a mineralized matrix by incubation with β-glycerophosphate. FGF-2 is also localized to sites of calcification within human atherosclerotic plaques. The expression of syndecan-4, a heparan sulfate proteoglycan which regulates FGF-2 signalling, is also increased in mineralizing VSMCs and co-localizes with FGF-2 in human calcified atherosclerotic plaques. Exogenous FGF-2 inhibits VSMC mineralization, and this inhibition is reduced when syndecan-4 expression is knocked-down using siRNA. Biochemical inhibition of FGFR signalling using a pan FGFR inhibitor (BGJ398) or knocking-down syndecan-4 expression in VSMCs using siRNA increases VSMC mineralization. These increases are prevented by inhibiting transforming growth factor-β (TGFβ) signalling with SB431542, suggesting cross-talk between FGF-2 and TGFβ signalling is crucial for the regulation of VSMC mineralization. Syndecan-4 can also regulate FGF-2 signalling directly via protein kinase Cα (PKCα) activation. Biochemical inhibition of PKCα activity using Gö6976, or siRNA-mediated suppression of PKCα expression increases VSMC mineralization; this increase is also prevented with SB431542. Finally, the ability of FGF-2 to inhibit VSMC mineralization is reduced when PKCα expression is knocked-down.

**Conclusion:**

This is the first demonstration that syndecan-4 promotes FGF-2 signalling, and in turn, suppresses VSMC mineralization by down-regulating TGFβ signalling. Our discoveries that FGF-2 and syndecan-4 expression is increased in mineralizing VSMCs and that PKCα regulates FGF-2 and TGFβ signalling in VSMCs suggests that the syndecan-4/FGF-2/TGFβ signalling axis could represent a new therapeutic target for vascular calcification.

## 1. Introduction

Vascular calcification is the formation of mineralized tissue, bone and/or cartilage within the vessel wall. Most patients with cardiovascular disease have some calcification, although it is most prevalent in patients with chronic kidney disease, type 2 diabetes mellitus and atherosclerosis.^1^^,^^2^ Calcification is not only highly prevalent in these diseases, but there is now substantial evidence that it contributes to the morbidity and mortality associated with these common conditions.^3^^,^^4^

Vascular calcification is an active cell-regulated process, involving the osteogenic differentiation of vascular smooth muscle cells (VSMCs), VSMC apoptosis, calcifying matrix vesicle/exosome release, and matrix mineralization.^5^^,^^6^ Existing approaches for the prevention of vascular calcification are limited; therefore, there is an urgent need to identify new therapeutic targets to treat this devastating pathology.

The fibroblast growth factors (FGFs) are a large family of secreted glycoproteins that can be classified as either paracrine- or endocrine-acting. Paracrine FGFs, such as FGF-2, are readily sequestered to the extracellular matrix by heparan sulfate proteoglycans (HSPGs) which limits their diffusion within the extracellular space. For signal propagation, the paracrine FGFs bind to a cell surface FGF-receptor (FGFR1-5) in a ternary complex consisting of FGF, FGFR and HPSGs leading to the activation of downstream signalling events via phospholipase Cγ and protein kinase C (PKC), Ras-Erk1/2 or PI3K-Akt.^7^

FGF-2 is a critical regulator of osteogenesis and bone development,^8^^,^^9^ although its role in this process is complex. Bone formation and mineralization are reduced in FGF-2-null mice.^10^^,^^11^ However, whilst short-term FGF-2 treatment stimulates matrix mineralization in calvarial osteoblasts^12^^,^^13^ and mesenchymal stem cells,^14^ continuous FGF-2 treatment inhibits mineralization by these cells.^12–16^ These studies suggest that FGF-2 is required to promote bone mineralization, but then must be down-regulated so mineralization can proceed.

Previous studies have shown that short-term FGF-2 treatment stimulates the expression of osteogenic markers in rat VSMCs.^17^ However, the potential role of FGF-2 in VSMC mineralization is currently unknown. Therefore, this study investigated whether FGF-2 regulates VSMC mineralization. We demonstrate that FGF-2/FGFR signalling plays an inhibitory role in this process by interacting with syndecan-4 and down-regulating transforming growth factor-β (TGFβ) signalling in VSMCs.

## 2. Methods

Detailed experimental protocols are in the Supplementary material online.

### 2.1 Reagents

Reagents were analytical grade and obtained from Sigma-Aldrich (UK) unless otherwise stated. Recombinant human FGF-2 (#100-18B) was from PeproTech (UK), recombinant human TGFβ1 (#240-B) from R&D Systems (UK), BGJ398 from Santa Cruz (USA), Gö6976 from Cell Signaling Technology (USA), and SB431542 from Sigma-Aldrich (UK). An equivalent volume of vehicle was used a control for each compound in experiments: 0.1% (w/v) bovine serum albumin (BSA) in 5 mM TRIS for FGF-2, dimethyl sulfoxide (DMSO) for BGJ398, Gö6976 and SB431542, and 0.1% (w/v) BSA in 4 mM HCl for TGFβ1. Antibodies to phosphorylated Smad2 (#3108), Smad2 (#5339), protein kinase Cα (PKCα, #2056), phosphorylated Akt (#4060), Akt (#9272), phosphorylated Erk1/2 (#4377), and Erk1/2 (#4695) were from Cell Signaling Technology (USA). Antibodies to syndecan-4 were from Santa Cruz (sc-12766) or Biovision, USA (#3644). Antibodies to FGF-2 (sc-79) were from Santa Cruz (USA), phosphorylated PKCα (07-790) from Merck Millipore (Germany), and β-actin (#A1978) from Sigma-Aldrich (UK).

### 2.2 Immunohistochemistry

Human atherosclerotic coronary arteries were used for the detection of FGF-2 (*n *=* *7) and syndecan-4 (*n *=* *5) by immunohistochemistry.^18^ Calcification was detected using von Kossa staining. Images were acquired using a *20x/0.80 Plan Apo* objective using the 3 D Histech Pannoramic 250 Flash II slide scanner. Human tissue was obtained with informed consent and with approval from the Local and National Research Ethics Committees (STH 16346, 12/NW/0036). This study conforms to the Declaration of Helsinki.

### 2.3 Cell culture

Bovine VSMCs were isolated from aortic explants obtained from a local abattoir, and routinely cultured in high glucose Dulbecco’s Modified Eagle Medium (DMEM) supplemented with 2 mM L-glutamine, 100 U/mL penicillin, 1.4 μM streptomycin, 1 mM sodium pyruvate, 1x non-essential amino acids and 10% (v/v) fetal calf serum (FCS), referred to as 10% FCS-DMEM. For mineralization assays, cells were cultured in 10% FCS-DMEM until confluent (day 0), and then in 10% FCS-DMEM and 3 or 5 mM β-glycerophosphate (β-GP) for up to 18 days.^19^ Controls were cultured without β-GP. Four preparations of uncloned VSMCs isolated from different animals were used for these studies; different batches of cells were used in independent experiments. Unless otherwise stated, *in vitro* studies used bovine VSMCs. Cells were used between passage 10–13.

Human coronary artery VSMCs were routinely cultured in medium 231 supplemented with smooth muscle growth supplement (Gibco, Life Technologies, UK). For mineralization assays, cells were cultured in medium 231 supplemented with smooth muscle growth supplement until confluent (day 0), and then with 5 mM β-GP and 0.9 mM calcium chloride for up to 40 days. The final concentration of calcium chloride in the human VSMC calcifying media was 2.5 mM. Controls were cultured without β-GP and additional calcium chloride. Two preparations of human VSMCs (passage 6–7) were used for these studies; different batches of cells were used in independent experiments.

### 2.4 Small interfering RNAs (siRNAs)

VSMCs were transfected with siRNAs against syndecan-4 (S459980, Ambion^®^, Life Technologies, UK) or PKCα (SI01965138, Qiagen, UK) using RNAiMAX (Invitrogen™, Life Technologies, UK). A random control siRNA (#1027281; Qiagen, UK) was the control. All siRNAs were used at a final concentration of 20 nM. For signalling assays, VSMCs were cultured for up to 7 days, with repeated siRNA transfections every 48–72 h. For mineralization assays, VSMCs were transfected twice with siRNA (with 48–72 h between transfections) prior to β-GP treatment. During β-GP treatment, siRNAs were removed after 4 h and fresh medium containing β-GP was added to the cells between transfections.

### 2.5 Alizarin red staining

Mineral deposition was confirmed by staining with 40 mM alizarin red (pH 4.1) and quantified by dye elution.^19^ The absorbance values for VSMC mineralization were: early mineralization (0.09–0.2), mid mineralization (0.21–0.6), and late mineralization (≥0.61).

### 2.6 Immunoblotting

Cell lysates were analysed for FGF-2, syndecan-4, phosphorylated Smad2, Smad2, phosphorylated PKCα, PKCα, phosphorylated Akt, Akt, phosphorylated Erk1/2, and Erk1/2 by immunoblotting.^20^ β-actin was the loading control. Immunoblots were quantified using ImageJ.

### 2.7 RNA isolation and quantitative polymerase chain reaction (qPCR)

RNA was isolated using the RNeasy Mini Kit (Qiagen) and cDNA synthesized using Taqman^®^ Reverse Transcription Reagents (Invitrogen™, Life Technologies). qPCR was performed using SYBR Green PCR master mix (Applied Biosystems, Life Technologies) and the CFX96 or CFX384 Real-Time PCR system (Bio-Rad, UK). Primer sequences are provided in the Supplementary material online. All samples were amplified in duplicate and averaged to produce one data-point. The expression of each gene was normalized to the reference genes [ribosomal protein L12 (RPL12) and peptidylprolyl isomerase A (PPIA)] using the comparative C_t_ method.

### 2.8 Statistical analysis

Data are presented as the mean ± standard error of the mean (SEM). Data were normalized where required using log_10_ and statistical comparisons were made using *t*-tests or one-way ANOVA. Data with two or more variables were analysed with a 2-way ANOVA. Where normality could not be confirmed, data were analysed using a Mann-Whitney *t*-test. A value of *P* < 0.05 was considered statistically significant.

## 3. Results

### 3.1 FGF-2 inhibits mineral deposition by VSMCs

**Figure 1:**
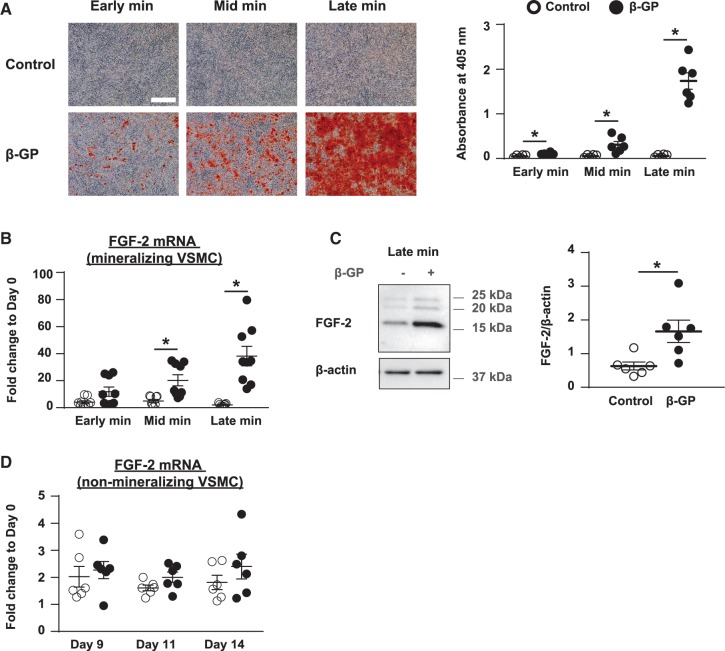
FGF-2 expression is up-regulated during VSMC mineralization. VSMCs (day 0) were incubated ± 3 mM β-GP for up to 18 days. (*A*) VSMCs were stained with alizarin red (bar = 500 µm) and mineral deposition quantified by dye elution (*n *=* *6 independent experiments). FGF-2 expression was measured using (*B*) qPCR (data expressed relative to day 0; *n *=* *9 independent experiments) and (*C*) immunoblotting of cell lysates (FGF-2 is expressed relative to β-actin; *n *=* *6 independent experiments). Molecular weight markers are shown. (*D*) Two different preparations of non-mineralizing VSMCs were cultured ± 5 mM β-GP from confluence (day 0) for up to 14 days. RNA was collected from cells at day 0, 9, 11, and 14. These time-points were chosen as they correspond to the time for mineralizing VSMCs (*1 A*) to reach early, mid or late mineralization. FGF-2 mRNA expression was measured using qPCR (data expressed relative to day 0; *n *=* *6 independent experiments). (A, D) Data are means ± SEM. (A, B, D) Data were normalized using log_10_ and analysed using 2-way ANOVA with Sidak post-hoc tests. (C) Data were analysed using a Mann-Whitney *t*-test. *P < 0.05.

FGF-2 plays an instructive role in osteogenesis,^12^^–^^14^ but the potential involvement of FGF-2 in vascular calcification was unknown. Therefore, to investigate FGF-2 expression during VSMC mineralization, a well-established *in vitro* model of vascular calcification was used.^19^ VSMCs deposited a mineralized matrix when cultured from confluence (day 0) in the presence of β-GP, and the extent of mineralization increased with time (*Figure 1A*). No mineralization was detected in controls cultured without β-GP (*Figure 1A*).

RNA and protein were isolated from VSMCs at specific time-points: early mineralization (days 9–10), mid mineralization (days 10–12), and late mineralization (days 12–18). RNA and protein were also isolated from VSMCs cultured without β-GP at these same time-points. FGF-2 mRNA (∼40-fold increase; *Figure 1B*) and protein (∼2.5-fold increase; *Figure 1C*) expression were significantly increased in β-GP-treated VSMCs at late mineralization when compared to controls at the same time-point. In contrast, FGF-2 expression was not increased in VSMC preparations that do not deposit a mineralized matrix in the presence of β-GP (*Figure 1D*) confirming that the changes observed in FGF-2 are either necessary for, or are a consequence of, VSMC mineralization and are not due to extended culture in the presence of β-GP.

**Figure 2:**
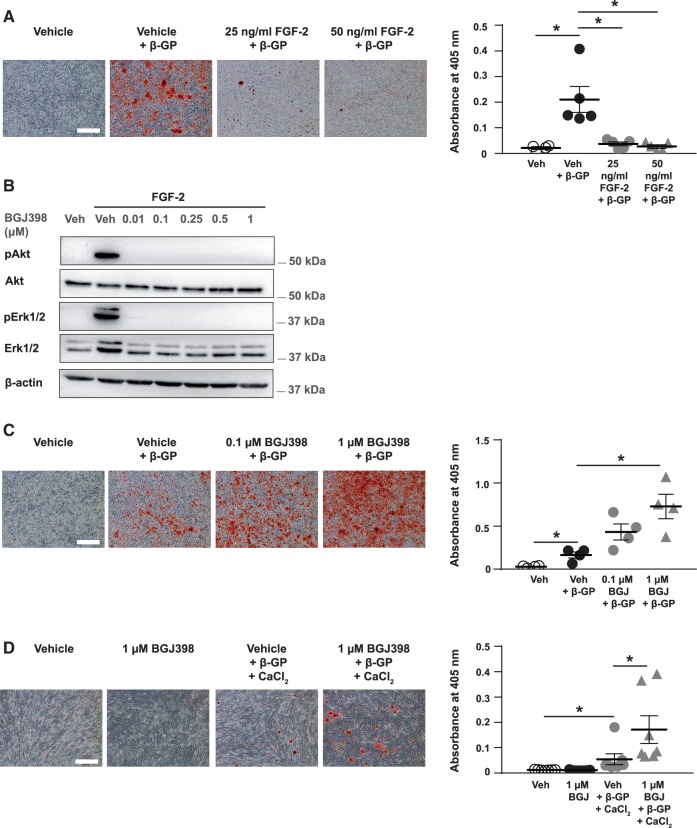
FGF-2/FGFR signalling regulates VSMC mineralization. (*A*) VSMCs were cultured with vehicle ± 3 mM β-GP, or FGF-2 (25 or 50 ng/mL) and 3 mM β-GP, stained with alizarin red (bar = 500 µm) and mineral deposition quantified (Vehicle, *n *=* *4 independent experiments; Vehicle and β-GP, 25 ng/mL FGF-2 and β-GP, 50 ng/mL FGF-2 and β-GP, *n *=* *5 independent experiments). (*B*) VSMCs were serum-starved for 2 h with vehicle (‘veh’) or BGJ398 (0.01–1 µM), and stimulated with FGF-2 for 5 min. Vehicle-treated VSMCs not stimulated with FGF-2 were controls. Cell lysates were immunoblotted for phosphorylated Akt (pAkt), total Akt, phosphorylated Erk1/2 (pErk1/2), and total Erk1/2. Two independent experiments were performed with two different concentrations of FGF-2 (25 and 50 ng/mL) with identical results; a representative immunoblot (50 ng/mL FGF-2) is shown. Molecular weight markers are shown. (*C*) VSMCs were cultured with vehicle ± 3 mM β-GP, or BGJ398 (0.1 or 1 μM) and 3 mM β-GP for up to 11 days, stained with alizarin red (bar = 500 µm) and mineral deposition quantified (*n *=* *4 independent experiments). (*D*) Human VSMCs were cultured with vehicle ± 5 mM β-GP and 0.9 mM calcium chloride, or 1 μM BGJ398 ± 5 mM β-GP and 0.9 mM calcium chloride, stained with alizarin red (bar = 500 µm) and mineral deposition quantified (*n *=* *7 independent experiments). (*A*, *C*, *D*) Data are means ± SEM. Data were normalized using log_10_ and analysed using a one-way ANOVA with Dunnett post-hoc tests. *P < 0.05.

To determine whether FGF-2 regulates VSMC mineralization, VSMCs were cultured with β-GP plus vehicle or FGF-2 (25 or 50 ng/mL). Exogenous FGF-2 significantly reduced β-GP-induced mineralization when compared to the vehicle and β-GP control (*Figure 2A*). The addition of FGF-2 at different time-points during the mineralization protocol (i.e. 0, 2, 4 or 6 days after addition of β-GP) also significantly reduced β-GP-induced mineralization in VSMCs compared to controls (see Supplementary material online, *Figure S1*), suggesting FGF-2 can also suppress matrix mineralization when added to cells which are primed to mineralize.

We next investigated the role of FGF-2-dependent fibroblast growth factor receptor (FGFR) signalling during VSMC mineralization using the pan-FGFR inhibitor BGJ398. BGJ398 markedly inhibited FGF-2-induced Akt and Erk1/2 phosphorylation in VSMCs (*Figure 2B*); BGJ398 also increased β-GP-induced VSMC mineralization compared to the vehicle and β-GP control (∼4.5-fold increase with 1 μM BGJ398; *Figure 2C*). This result was verified in mineralizing human VSMCs (*Figure 2D*). BGJ398 did not induce VSMC mineralization in the absence of raised phosphate levels (see Supplementary material online, *Figure S2A*), nor did it induce mineralization in preparations of VSMCs that do not mineralize in the presence of β-GP (see Supplementary material online, *Figure S2B*), suggesting that inhibition of FGFR signalling does not drive VSMC mineralization on its own, but it accelerates mineralization in VSMCs that are already primed to mineralize.

### 3.2 FGF/TGF**β** cross-talk regulates mineral deposition by VSMCs

The above results demonstrate FGF-2 and FGFR signalling reduce matrix mineralization in VSMCs, but how FGF-2 mediates this effect was unknown. Recent studies have shown that inhibiting FGF signalling increases TGFβ signalling in VSMCs.^21^^,^^22^ As TGFβ1 accelerates mineral deposition by calcifying vascular cells,^23^ we next investigated the relationship between FGF and TGFβ signalling in VSMC mineralization.

**Figure 3:**
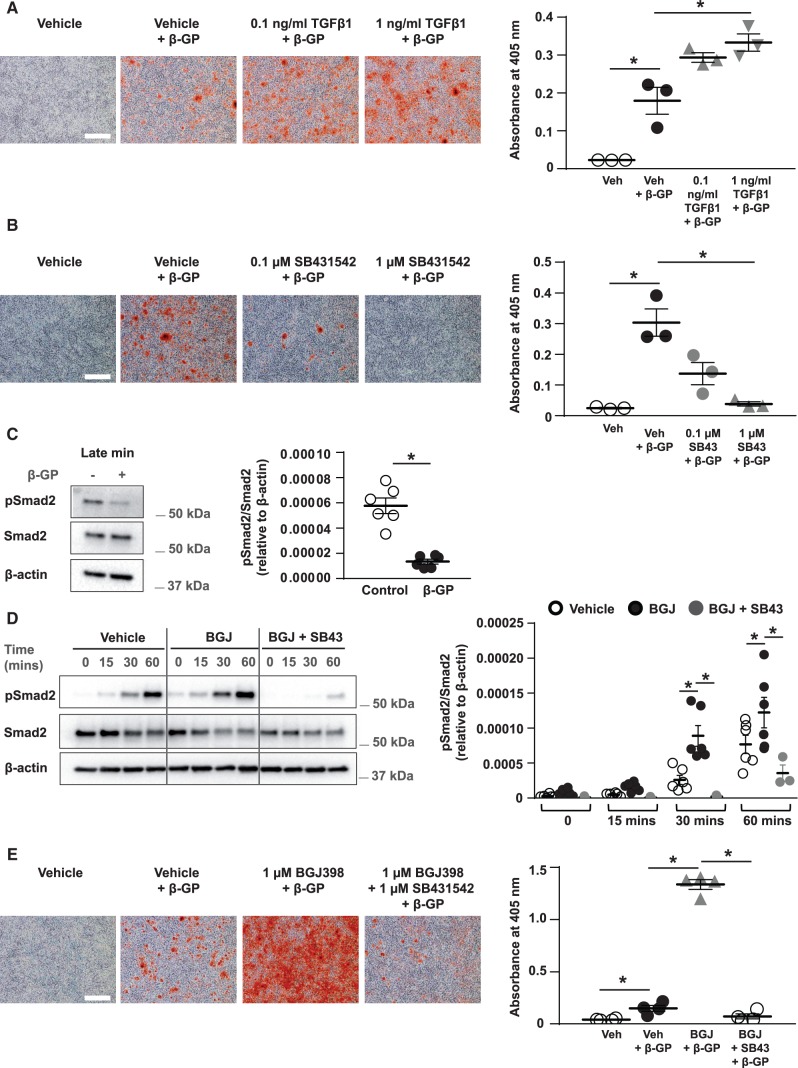
FGFR/TGFβ cross-talk regulates mineral deposition by VSMCs. (*A*) VSMCs were cultured with vehicle ± 3 mM β-GP, or TGFβ1 (0.1 or 1 ng/mL) and 3 mM β-GP, stained with alizarin red (bar = 500 µm) and mineral deposition quantified (*n *=* *3 independent experiments). (*B*) VSMCs were cultured with vehicle ± 3 mM β-GP, or SB431542 (0.1 or 1 μM) and 3 mM β-GP, stained with alizarin red (bar = 500 µm) and mineral deposition quantified (*n *=* *3 independent experiments). (*C*) VSMCs were incubated ± 3 mM β-GP for up to 18 days. Cell lysates were isolated at late VSMC mineralization and immunoblotted for phosphorylated Smad2 (pSmad2), total Smad2, and β-actin on the same membrane. Molecular weight markers are shown. The pSmad2/Smad2 ratio is expressed relative to β-actin (*n *=* *6 independent experiments). (*D*) VSMCs were serum-starved for 2 h (‘0’) with vehicle, BGJ398 (1 µM), or with BGJ398 (1 µM) and SB431542 (1 µM), stimulated with 0.5 ng/mL TGFβ1 for 15, 30 or 60 min and immunoblotted for phosphorylated Smad2 (pSmad2), total Smad2, and β-actin on the same membrane. Molecular weight markers are shown. The pSmad2/Smad2 ratio is expressed relative to β-actin (vehicle, BGJ398, *n *=* *6 independent experiments*;* BGJ398 with SB431542*, n = *3 independent experiments). (*E*) VSMCs were cultured with vehicle ± 5 mM β-GP, 5 mM β-GP and BGJ398 (1 µM), or with 5 mM β-GP, BGJ398 (1 µM) and SB431542 (1 µM), stained with alizarin red (bar = 500 µm) and mineral deposition quantified (*n *=* *4 independent experiments). (*A*–*E*) Data are means ± SEM. (*A*, *B*, *E*) Data were normalized using log_10_ and analysed using a one-way ANOVA with Dunnett post-hoc tests. (*C*) Data were analysed using a Mann-Whitney *t*-test. (*D*) Data were analysed using a 2-way ANOVA with Sidak post-hoc tests. *P < 0.05.

To confirm TGFβ signalling regulates VSMC mineralization, VSMCs were cultured with β-GP plus vehicle or TGFβ1 (0.1 or 1 ng/mL). Exogenous TGFβ1 significantly increased β-GP-induced VSMC mineralization when compared to the vehicle and β-GP control (*Figure 3A*). In contrast, inhibiting endogenous TGFβ signalling using the type 1 TGFβ receptor (TGFβR1) kinase inhibitor, SB431542 (0.1 or 1 µM), significantly reduced VSMC mineralization when compared to the vehicle and β-GP control (*Figure 3B*).

TGFβR activation leads to Smad2 phosphorylation.^21^^,^^22^ Previous studies have shown that decreased Smad2 phosphorylation co-localizes with increased FGFR1 phosphorylation in the medial layer of atherosclerotic human coronary arteries.^22^ In the late stages of matrix mineralization, Smad2 phosphorylation was significantly reduced in β-GP-treated VSMCs when compared to controls at the same time-point (four-fold decrease; *Figure 3C*). This decrease in Smad2 phosphorylation coincided with increased FGF-2 expression in β-GP-treated VSMCs (compare *Figures 1B, 1C* and*3C*).

To confirm FGF regulates TGFβ signalling in VSMCs, FGFR signalling was inhibited using BGJ398 and cells were incubated with TGFβ1 for up to 60 min. TGFβ1-induced Smad2 phosphorylation was significantly increased in VSMCs treated with BGJ398 after 30 and 60 min (*Figure 3D*); this increase was prevented by co-incubation with SB431542 (*Figure 3D*). The relationship between FGF and TGFβ signalling in matrix mineralization was also studied by incubating VSMCs with both inhibitors. As before, BGJ398 significantly increased β-GP-induced VSMC mineralization compared to the vehicle and β-GP control (*Figure 3E*); this increase was prevented by co-incubation with SB431542 (*Figure 3E*). Together these results suggest FGFR inhibition increases matrix mineralization by up-regulating TGFβ signalling in VSMCs.

### 3.3 Syndecan-4 expression co-localizes with FGF-2 in calcified vessels

Syndecan-4 is a transmembrane HSPG that functions as an adhesion receptor and growth factor co-receptor, eliciting signals in response to the extracellular microenvironment.^24^^,^^25^ Atherosclerotic plaque susceptibility is increased in syndecan-4/low-density lipoprotein receptor double knock-out mice fed a high-fat diet^26^; but its role in vascular calcification was unknown. As syndecan-4 is a critical regulator of FGF-2 signalling,^27^^,^^28^ we next investigated the potential involvement of syndecan-4 in VSMC mineralization, and examined whether syndecan-4 regulates FGF-2/TGFβ signalling in this process.

**Figure 4:**
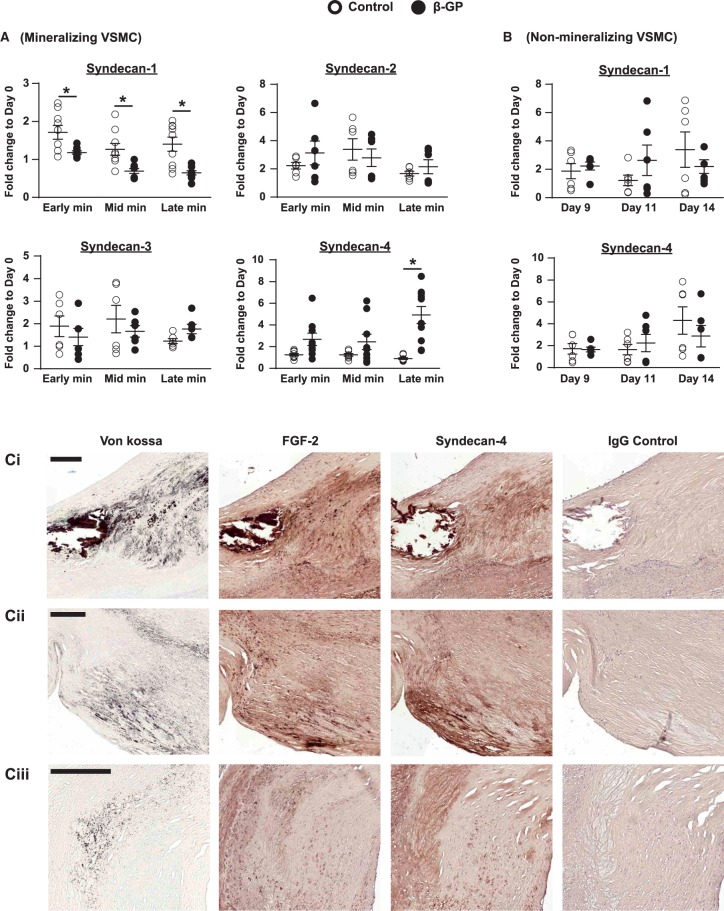
Syndecan-4 expression co-localizes with FGF-2 in vascular calcification. (*A*) VSMCs (day 0) were incubated ± 3 mM β-GP for up to 14 days. The mRNA levels of syndecan-1-4 were measured using qPCR (as in *Figure 1B; n *=* *9 independent experiments for syndecan-1 and syndecan-4; *n *=* *6 independent experiments for syndecan-2 and syndecan-3). (*B*) Two different preparations of non-mineralizing VSMCs were cultured ± 5 mM β-GP from confluence (day 0) for up to 14 days and the expression levels of syndecan-1 and syndecan-4 were measured using qPCR (as in *Figure 1 D; n *=* *6 independent experiments). (*A*, *B*) Data are means ± SEM. Data were normalized using log_10_ and analysed using 2-way ANOVA with Sidak post-hoc tests. *P < 0.05. (*C*) Atherosclerotic coronary artery specimens stained with von Kossa’s reagent for calcification (black), anti-FGF-2 antibody, anti-syndecan-4 antibody, or rabbit IgG. FGF-2 and syndecan-4 (brown) localize to areas adjacent to, or within, calcified regions in atherosclerotic plaques ((Ci), plaque with extensive calcification, patient 167; (Cii), plaque with more diffuse calcification, patient 167; (Ciii) plaque with spicules of calcification, patient 161). (Ci-iii) Rabbit IgG controls are negative. Bars = 200 µm.

Syndecan-4 mRNA expression was markedly increased in β-GP-treated VSMCs, with a ∼five-fold increase at late mineralization compared to the same time-point controls (*Figure 4A*). This increase in syndecan-4 mRNA expression coincided with increased FGF-2 expression and decreased Smad2 phosphorylation in β-GP-treated VSMCs (compare *Figures 1B, 1C, 3C* and *4A*). In contrast, syndecan-1 expression was significantly decreased in β-GP-treated VSMCs compared to controls, with a ∼two-fold decrease at late mineralization (*Figure 4A*). No significant changes were detected in syndecan-2 or syndecan-3 mRNA expression (*Figure 4A*). Furthermore, syndecan-1 and syndecan-4 mRNA expression did not change when VSMC preparations which do not deposit a mineralized matrix in the presence of β-GP were analysed (*Figure 4B*).

Syndecan-4 and FGF-2 expression in human atherosclerotic arteries was also examined. FGF-2 and syndecan-4 staining was localized to areas directly adjacent to, and within, calcified regions of atherosclerotic arteries (representative images of three atherosclerotic lesions from two different donors are shown in *Figure 4Ci–iii*). No staining was observed in the rabbit IgG controls (*Figure 4Ci–iii*).

### 3.4 FGF-2 inhibits mineral deposition via syndecan-4

**Figure 5:**
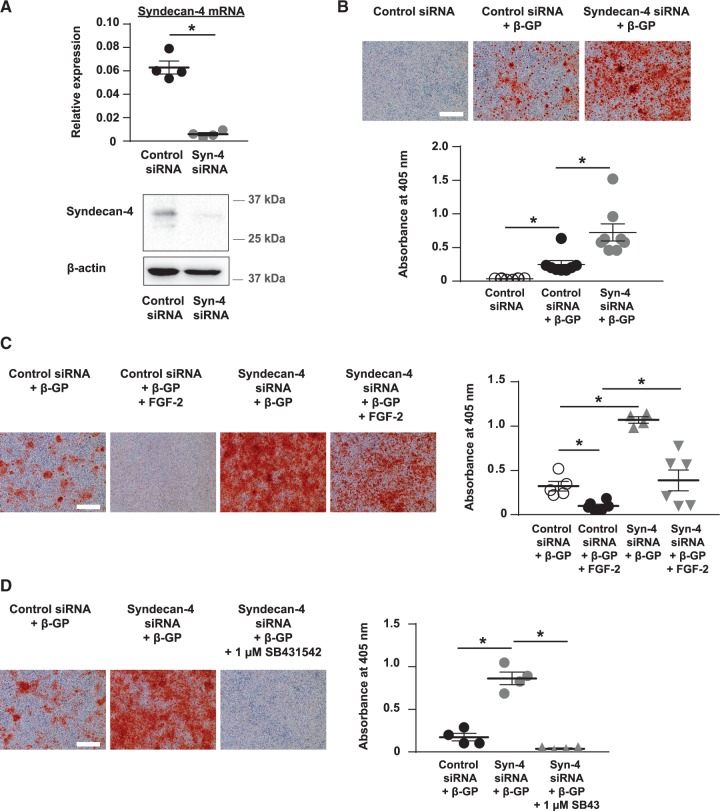
Knocking-down syndecan-4 rescues the inhibitory effect of FGF-2 on VSMC mineralization. (*A*) Syndecan-4 expression was knocked-down in VSMCs using siRNA and confirmed using qPCR (upper panel) and immunoblotting (lower panel) (*n *=* *4 independent experiments). (*B*) VSMCs transfected with syndecan-4 siRNA were cultured with 3 mM β-GP for up to 9 days. Control siRNA-treated VSMCs cultured ± 3 mM β-GP were controls. Cells were stained with alizarin red (bar = 500 µm) and mineral deposition quantified (*n *=* *8 independent experiments). Results were verified using a different siRNA oligonucleotide sequence to target syndecan-4 in VSMCs (not shown). (*C*) Control siRNA and syndecan-4 siRNA-treated VSMCs were cultured with 5 mM β-GP and vehicle or FGF-2, stained with alizarin red (bar = 500 µm) and mineral deposition quantified (Control siRNA/β-GP, Control siRNA/β-GP/FGF-2 and Syndecan-4 siRNA/β-GP/FGF-2, *n *=* *6 independent experiments; Syndecan-4 siRNA/β-GP, *n *=* *4 independent experiments). (*D*) Syndecan-4 siRNA-treated VSMCs were cultured with 5 mM β-GP and vehicle or SB431542 (1 µM). Control siRNA-treated VSMCs cultured with 5 mM β-GP and vehicle were controls. Cells were stained with alizarin red (bar = 500 µm) and mineral deposition quantified (*n *=* *4 independent experiments). (*A*–*D*) Data are means ± SEM. (*A*) Data were normalized using log_10_ and analysed using a *t*-test. (*B*, *D*) Data were normalized using log_10_ and analysed using a one-way ANOVA with Dunnett post-hoc tests. (*C*) Data were normalized using log_10_ and analysed using a one-way ANOVA with Tukey post-hoc tests. *P < 0.05.

To determine the role of syndecan-4 in VSMC mineralization, siRNA was used to knock-down syndecan-4 expression (*Figure 5A*). Syndecan-4 knock-down significantly increased β-GP-induced VSMC mineralization compared to control siRNA-treated cells cultured with β-GP (*Figure 5B*). Knocking-down syndecan-4 expression in VSMCs cultured in control media did not induce matrix mineralization on its own (see Supplementary material online, *Figure S3A*). Furthermore, knocking-down syndecan-4 expression in a preparation of VSMCs that do not mineralize in the presence of β-GP did not induce matrix mineralization (see Supplementary material online, *Figure S3B*).

Syndecan-4 knock-down or biochemical inhibition of FGF-2-dependent FGFR signalling increases VSMC mineralization, suggesting syndecan-4 and FGF-2 expression are increased in mineralizing VSMCs to prevent further calcification. To investigate the link between FGF-2 and syndecan-4 in regulating VSMC mineralization, control siRNA-, and syndecan-4 siRNA-transfected VSMCs were cultured with vehicle or FGF-2 in the presence of β-GP. As before, FGF-2 inhibited mineralization whereas syndecan-4 knock-down markedly increased mineralization (*Figure 5C*). Furthermore, the inhibitory effect of FGF-2 on matrix mineralization was partially prevented by knocking-down syndecan-4 expression (*Figure 5C*). However, FGF-2 was still able to inhibit mineralization in syndecan-4 knock-down VSMCs (*Figure 5C*), suggesting FGF-2 can also signal independently of its co-receptor, syndecan-4.

Inhibiting FGFR signalling increases TGFβ signalling in VSMCs (*Figure 3D*) and inhibiting TGFβ signalling prevents FGFR inhibition from increasing VSMC mineralization (*Figure 3E*). Therefore, to determine if TGFβ signalling is also responsible for the increased matrix mineralization in syndecan-4 knock-down VSMCs, syndecan-4 siRNA-transfected VSMCs were cultured with β-GP and vehicle or SB431542 (1 μM). Control siRNA-transfected VSMCs cultured with β-GP were used as controls. Knocking-down syndecan-4 expression in VSMCs significantly increased β-GP-induced matrix mineralization; this increase was prevented by co-incubation with SB431542 (*Figure 5D*). These results suggest syndecan-4 and FGF-2 both suppress matrix mineralization by down-regulating TGFβ signalling in VSMCs.

### 3.5 PKCα signalling regulates mineral deposition

**Figure 6:**
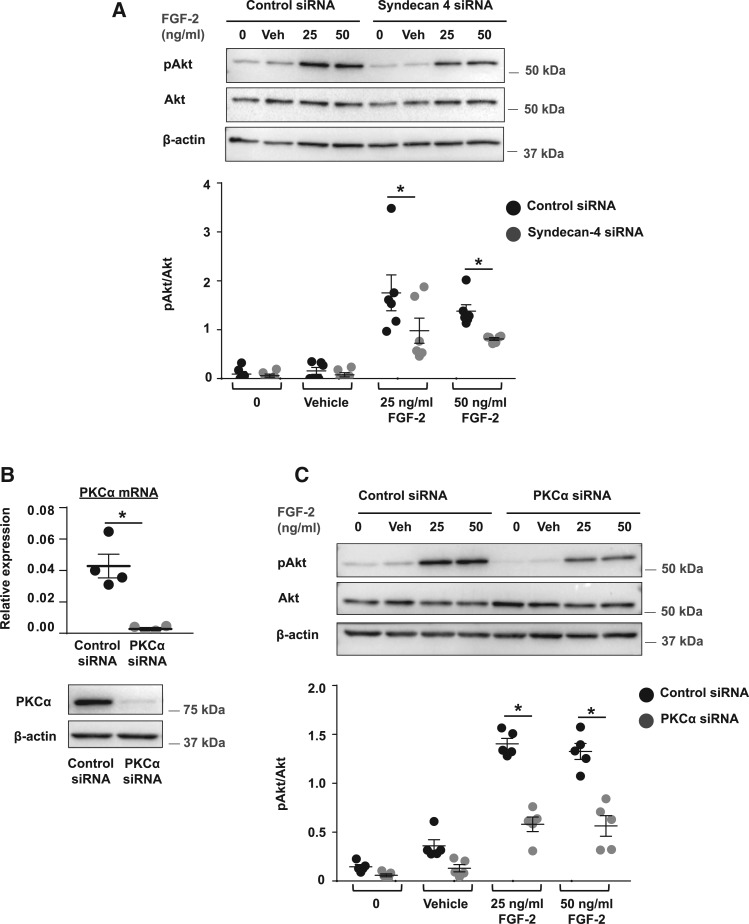
Syndecan-4 and PKCα regulate FGF-2/Akt signalling in VSMCs. (*A*) Control and syndecan-4 siRNA-treated VSMCs were serum-starved for 2 h (0) and stimulated with vehicle or FGF-2 (25 and 50 ng/mL) for 5 min. Cell lysates were immunoblotted for phosphorylated Akt (pAkt) and total Akt; β-actin was the loading control (*n *=* *6 independent experiments). Molecular weight markers and the pAkt/Akt ratio are shown. (*B*) PKCα expression was knocked-down in VSMCs using siRNA and confirmed using qPCR (upper panel) and immunoblotting (lower panel) (*n *=* *4 independent experiments). (*C*) FGF-2/Akt signalling assays were performed with control and PKCα siRNA-treated VSMCs as described in (*A*) (*n *=* *5 independent experiments). Molecular weight markers and the pAkt/Akt ratio are shown. (*A*–*C*) Data are means ± SEM. (*A*, *C*) Data were analysed using a 2-way ANOVA with Sidak post-hoc tests. (*B*) Data were normalized using log_10_ and analysed using a *t*-test. *P < 0.05.

**Figure 7:**
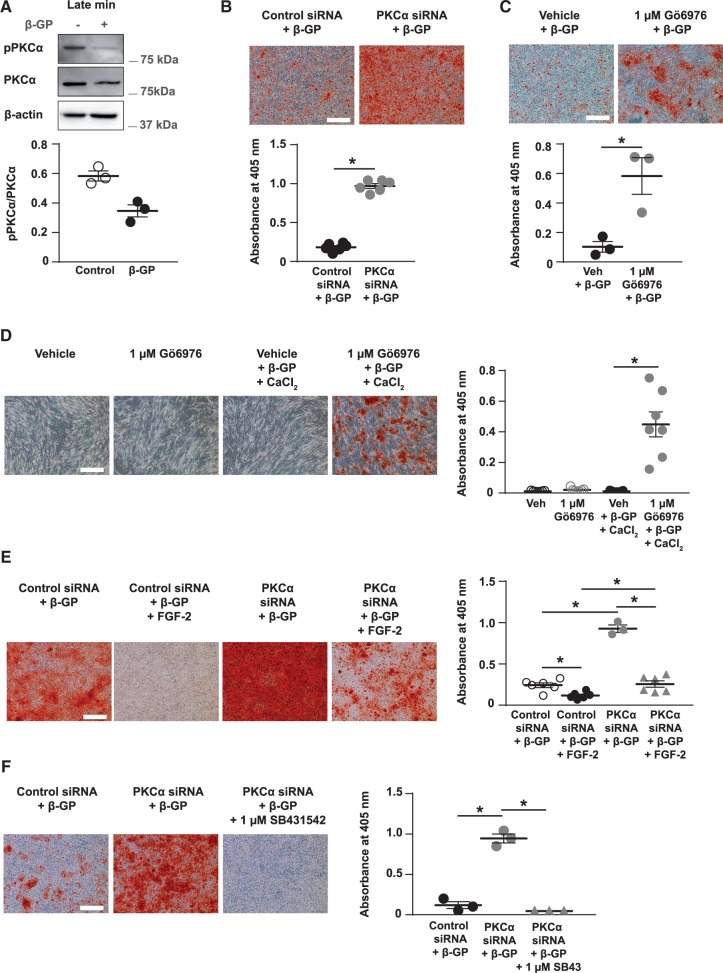
Inhibiting PKCα activity with Gö6976, or knocking-down PKCα expression using siRNA, increases VSMC mineralization. (*A*) VSMCs (day 0) were incubated ± 3 mM β-GP for up to 14 days. Cell lysates were isolated at late VSMC mineralization and immunoblotted for PKCα phosphorylation (pPKCα), total PKCα, and β-actin on the same membrane (*n *=* *3 independent experiments). Molecular weight markers and the pPKCα/PKCα ratio are shown. (*B*) VSMCs transfected with control siRNA or PKCα siRNA were cultured with 3 mM β-GP for up to 9 days, stained with alizarin red (bar = 500 µm) and mineral deposition quantified (*n *=* *6 independent experiments). (*C*) VSMCs were cultured with 3 mM β-GP and vehicle or 1 μM Gö6976 for up to 11 days, stained with alizarin red (bar = 500 µm) and mineral deposition quantified (*n *=* *3 independent experiments). (*D*) Human VSMCs were cultured with vehicle ± 5 mM β-GP and 0.9 mM calcium chloride, or 1 μM Gö6976 ± 5 mM β-GP and 0.9 mM calcium chloride, stained with alizarin red (bar = 500 µm) and mineral deposition quantified (*n *=* *7 independent experiments). (*E*) Control siRNA and PKCα siRNA-treated VSMCs were cultured with 5 mM β-GP and vehicle or FGF-2, stained with alizarin (bar = 500 µm) and mineral deposition quantified (Control siRNA/β-GP, Control siRNA/β-GP/FGF-2, and PKCα siRNA/β-GP/FGF-2, *n *=* *6 independent experiments; PKCα siRNA/β-GP, *n *=* *3 independent experiments). (*F*) PKCα siRNA-treated VSMCs were cultured with 5 mM β-GP and vehicle or SB431542 (1 µM). Control siRNA-treated VSMCs cultured with 5 mM β-GP and vehicle were used as controls. Cells were stained with alizarin red (bar = 500 µm) and mineral deposition quantified (*n *=* *3 independent experiments). (*A*–*F*) Data are means ± SEM. (*B*, *C*) Data were normalized using log_10_ and analysed using a *t*-test. (*D*, *F*) Data were normalized using log_10_ and analysed using a one-way ANOVA with Dunnett post-hoc tests. (*E*) Data were normalized using log_10_ and analysed using a one-way ANOVA with Tukey post-hoc tests. *P < 0.05.

The cytoplasmic domain of syndecan-4 interacts with, and activates, PKCα^29^^,^^30^ and the syndecan-4/PKCα complex regulates FGF-2-induced Akt phosphorylation in endothelial cells.^27^^,^^28^ To investigate the link between FGF-2, syndecan-4 and PKCα in VSMCs, syndecan-4 and PKCα were knocked-down using siRNA and the cells were incubated with vehicle or FGF-2 for 5 min and down-stream signalling via Akt assessed. FGF-2-induced Akt phosphorylation was reduced in syndecan-4 siRNA-transfected VSMCs (*Figure 6A*) and PKCα siRNA-transfected VSMCs (*Figure 6Band*C). Western blotting also revealed a trend towards decreased phosphorylated PKCα during the late stages of VSMC mineralization (*Figure*7A). Therefore, we next investigated whether PKCα regulates FGF-2/TGFβ signalling and mineralization in VSMCs.

Knocking-down PKCα with siRNA (*Figure 7B)* or inhibiting PKCα activity with Gö6976 (1 μM) (*Figure 7C*) significantly increased β-GP-induced VSMC mineralization compared to the relevant controls. Gö6976 also increased mineralization in human VSMCs (*Figure 7D*). Knocking-down PKCα expression in VSMCs cultured in control media (see Supplementary material online, *Figure S3A*), or culturing VSMCs in control media with Gö6976 (see Supplementary material online, *Figure S3C*) did not induce matrix mineralization. Also Gö6976 did not induce mineralization in a preparation of VSMCs that do not mineralize in the presence of β-GP (see Supplementary material online, *Figure S3D*). These results suggest that loss or inhibition of PKCα is not a driver of VSMC mineralization *per se*, but it accelerates mineralization in VSMCs which are already primed to mineralize.

To further define the link between FGF-2/syndecan-4 and PKCα in regulating VSMC mineralization, control siRNA- and PKCα siRNA-transfected VSMC were cultured with vehicle or FGF-2 in the presence of β-GP. As before, FGF-2 reduced mineralization whereas PKCα knock-down markedly increased mineralization (*Figure 7E*). PKCα knock-down reduced the inhibitory effect of FGF-2 on mineralization (*Figure 7E*), suggesting the FGF-2/syndecan-4 signalling axis may, at least in part, regulate VSMC mineralization via PKCα. FGF-2 was still able to inhibit mineralization in PKCα knock-down VSMCs (*Figure 7E*), suggesting that FGF-2 can also signal via other downstream signalling pathways.

To determine if increased TGFβ signalling mediates the increased mineralization in PKCα knock-down VSMCs, PKCα siRNA-transfected VSMCs were cultured with β-GP and vehicle or SB431542 (1 μM). Control siRNA-transfected VSMCs cultured with β-GP were controls. SB431542 prevented PKCα knock-down from increasing β-GP-induced matrix mineralization in VSMCs (*Figure 7F*), suggesting loss of PKCα increases matrix mineralization by up-regulating TGFβ signalling in VSMCs.

## 4. Discussion

**Figure 8:**
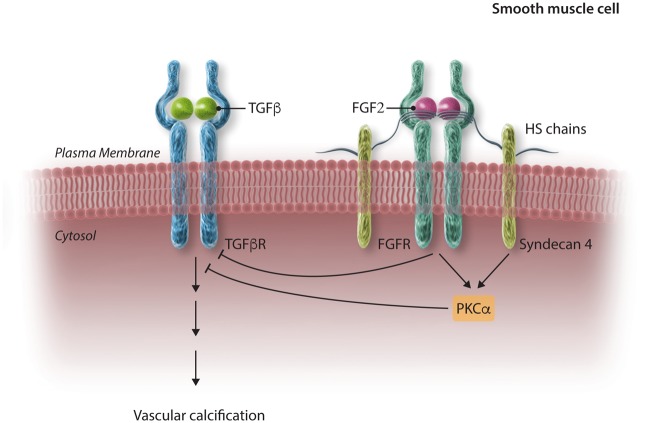
Schematic diagram demonstrating how cross-talk between syndecan-4, FGF-2, and TGFβ signalling may regulate calcification. Not to scale.

We demonstrate the expression of FGF-2 and its co-receptor, syndecan-4, are increased in mineralizing VSMCs and at sites of calcification in human atherosclerotic plaques, and that biochemical inhibition of FGFR signalling or knocking-down syndecan-4 expression increases VSMC mineralization. Importantly, syndecan-4 is, at least in part, responsible for the inhibition of VSMC mineralization by FGF-2, suggesting syndecan-4 expression is increased in mineralizing VSMCs to maintain FGF-2 signalling. We also show syndecan-4 and FGF-2 signalling suppress the deposition of a mineralized matrix by down-regulating TGFβ signalling. Finally, we demonstrate that PKCα, which is activated in a cytoplasmic domain-dependent manner by syndecan-4,^29^^,^^30^ regulates FGF-2/TGFβ signalling and mineralization in VSMCs. Together, these results demonstrate a novel feedback mechanism whereby mineralizing VSMCs increase FGF-2 and syndecan-4 expression and down-regulate TGFβ signalling to prevent more extensive calcification (*Figure 8*).

This is the first demonstration that FGF-2 expression is increased in mineralizing VSMCs *in vitro*, and that FGF-2 is localized to calcified regions of human atherosclerotic plaques. Consistent with these findings, FGF-2 is expressed adjacent to calcified regions in valve leaflets.^31^ FGF-2 mRNA expression is also increased during the osteogenic differentiation and mineralization of osteoprogenitors,^32^ and is expressed at sites of bone formation *in vivo*.^33^ FGF-2 plays a complex role in osteoblast mineralization, with its effects dependent on the timing and duration of signalling.^12^^–^^14^ Consistent with these studies, we show that continuous FGF-2 treatment inhibits β-GP-induced VSMC mineralization. The addition of FGF-2 after the commencement of β-GP treatment also reduces VSMC mineralization, supporting the suggestion that increases in FGF-2 expression during late mineralization may put a ‘brake’ on this process. Indeed, FGF-2 stimulates the osteogenic potential of calvarial osteoblasts and mesenchymal stem cells during the early stages of differentiation, but must then be down-regulated for mineralization to proceed.^12^^–^^14^

It is well established that syndecan-4 is a critical regulator of FGF-2 signalling,^27^^,^^28^ but it is currently unknown how the transcription of syndecan-4 and FGF-2 is regulated in mineralizing VSMCs. Previous studies have reported that FGF-2 synergizes with Runx2 to enhance syndecan-4 mRNA expression in calvarial osteoblasts,^34^ but we found that 24-h FGF-2 treatment has no effect on syndecan-4 mRNA expression in VSMCs (see Supplementary material online, *Figure S4*). It is possible, therefore, that raised levels of Runx2 are required for FGF-2 to induce syndecan-4 expression in these cells e.g. as observed during VSMC osteogenic differentiation and mineralization.

A role for TGFβ1 in vascular calcification was first suggested by Watson *et al.* who reported TGFβ1 increased mineralized nodule formation in bovine calcifying vascular cells.^23^ More recent studies have shown that inhibition of TGFβR1 using SB431542 inhibits VSMC mineralization.^35^^,^^36^ We also show that TGFβ1 accelerates mineral deposition by bovine VSMCs, whereas SB431542 inhibits it. Furthermore, we show FGF-2 expression is increased in mineralizing VSMCs and TGFβ signalling is concomitantly reduced to minimize further calcification. Previous studies in VSMCs have shown that suppressing FGF signalling results in reduced *let-7* microRNA, leading to increased TGFβR1 receptor expression and TGFβ signalling activation.^22^ It is therefore possible that FGF-2/TGFβ cross-talk may also be mediated via *let-7* microRNA in mineralizing VSMCs.

Several studies have suggested PKCα normally acts to suppress bone formation.^37^^,^^38^ Consistent with a previous study in mouse VSMCs,^39^ we show that inhibiting PKCα activity with Gö6976 or knocking-down PKCα expression increases VSMC mineralization. Moreover, we demonstrate that this increase in mineralization is prevented by inhibiting TGFβR1 signalling. The crucial role of PKCα in regulating mineralization is further highlighted by our demonstration that knocking-down PKCα reduces the ability of FGF-2 to inhibit VSMC mineralization. Over-expressing PKCα in an osteoblastic cell line reduces alkaline phosphatase activity and the expression of osteogenic marker genes in these cells^37^; however, the effects of over-expressing PKCα on osteoblast or VSMC mineralization are unknown. As PKCα is downstream of FGF-2/syndecan-4, a possible focus for therapeutic targeting in vascular calcification may be the modulation of PKCα activation/signalling in VSMCs.

A potential limitation of our study is that the signalling data were obtained following short-term incubation of the VSMCs with growth factors and/or inhibitors; whereas the mineralization data were obtained following incubation of the cells with these same reagents for up to 14 days. However, although caution should be taken when extrapolating between these two sets of data, our study clearly demonstrates that signalling and mineralization are both affected by these treatments.

Whilst our results indicate an important role for syndecan-4 in regulating FGF-2/TGFβ signalling during VSMC mineralization, other PGs could also regulate these signalling pathways in this process. Indeed, our data show the expression levels of several other PGs are modulated during VSMC mineralization (see Supplementary material online, *Figure S5*). For example, glypican-4 expression is also up-regulated in β-GP-treated VSMCs (see Supplementary material online, *Figure S5*). Glypican-4 binds FGF-2^40^ and may therefore also affect FGF-2/FGFR signalling in VSMCs, although glypican-4 wouldn’t directly activate PKCα. Decorin has also been shown to promote mineralization by increasing TGFβ signalling in VSMCs.^35^ Future studies could, therefore, determine whether these PGs also regulate FGF-2 and TGFβ cross-talk during VSMC mineralization.

In conclusion, our study has identified a novel potential therapeutic target pathway in the control of vascular disease. We highlight syndecan-4/FGF-2/TGFβ signalling as a critical regulator of VSMC mineralization. Intriguingly, both syndecan-4 and FGFR signalling appear to be important in this process. It remains to be determined whether syndecan-4 and FGFR regulate mineralization in convergent or parallel pathways. It is possible that syndecan-4 may act to prevent excessive mineralization via two mechanisms: (a) interacting as a co-receptor for FGF-2 and inducing down-stream signalling via FGFR and (b) via interaction with PKCα. These pathways may then coalesce to suppress mineralization induced by TGFβ. Although this dual activity of syndecan-4 is well established in other systems (e.g. during neural induction^41^) its role here is of particular importance given the current need for novel drugs to treat vascular disease.

## Supplementary material

Supplementary material is available at *Cardiovascular Research* online.

## Supplementary Material

Supplementary Data

Supplementary Data

Supplementary Data

Supplementary Data

Supplementary Data

Supplementary Data

Supplementary Data
