# Supplementary figures and images for: Regulation of vascular smooth muscle cell calcification by syndecan-4/FGF-2/PKCα signalling and cross-talk with TGFβ
Source: Cardiovasc Res. 2017 Sep 6;113(13):1639–52. doi: 10.1093/cvr/cvx178 (PMC5852548; doi:10.1093/cvr/cvx178)

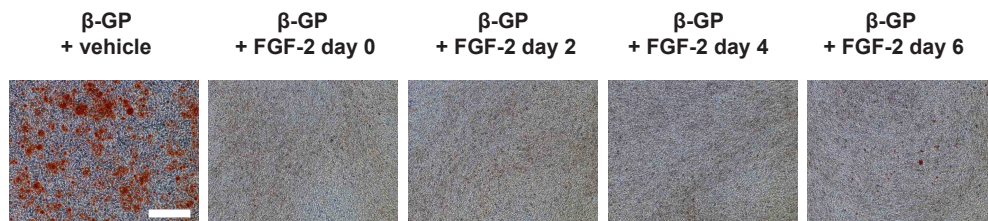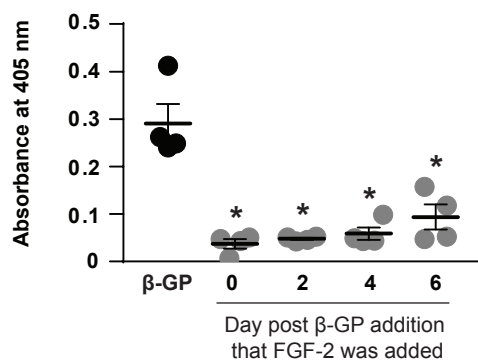

Supplement: Supplementary Data [file cvx178_figure_s1.pdf]

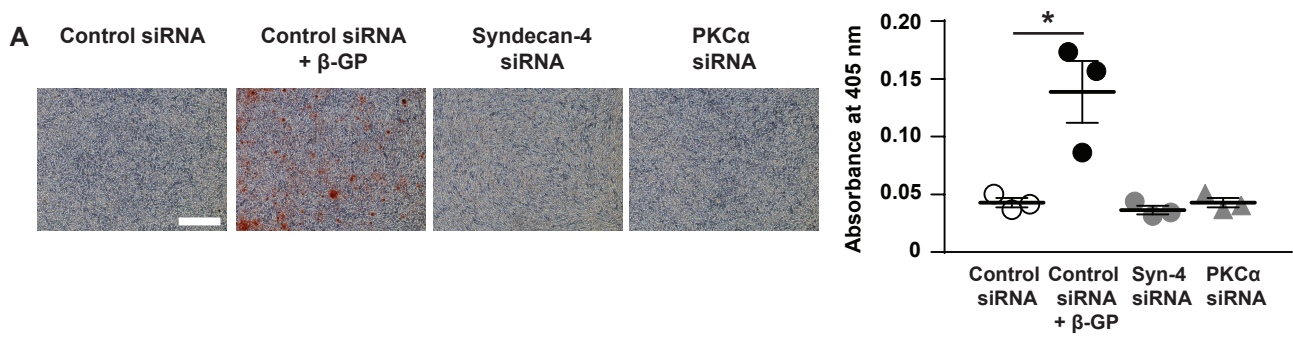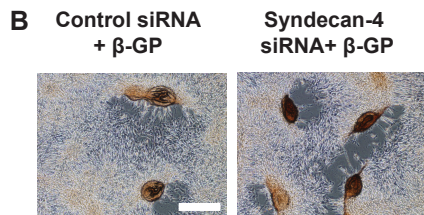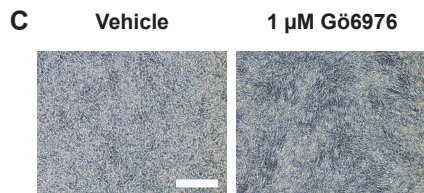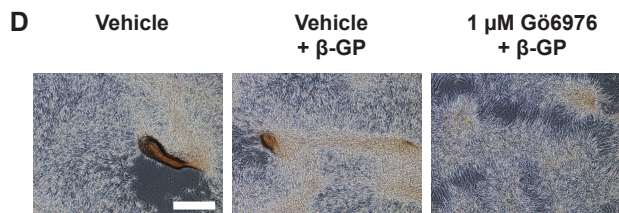

Supplement: Supplementary Data [file cvx178_figure_s3.pdf]

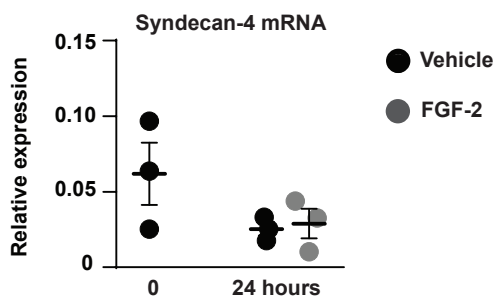

Supplement: Supplementary Data [file cvx178_figure_s4.pdf]

○ Control    ●  $\beta$ -GP

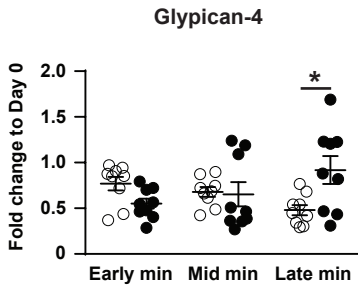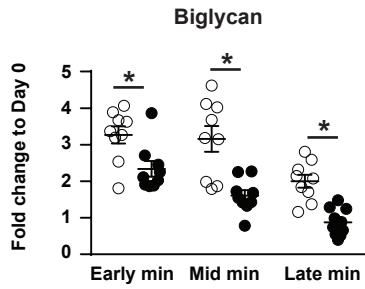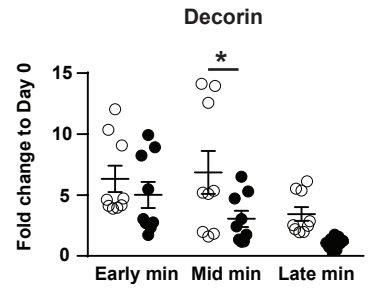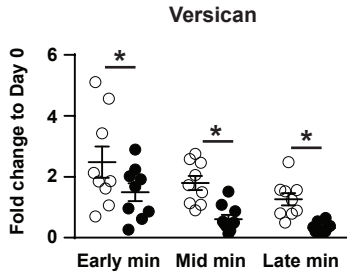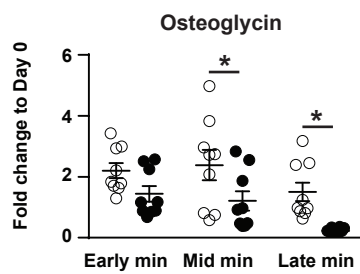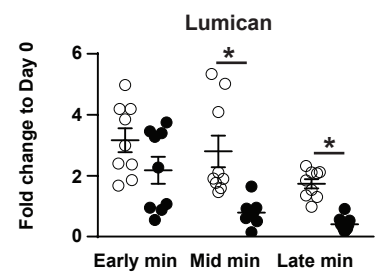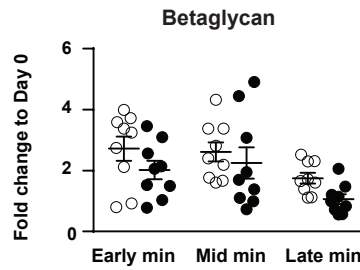

Supplement: Supplementary Data [file cvx178_figure_s5.pdf]
