# Supplementary material for: Regulation of vascular smooth muscle cell calcification by syndecan-4/FGF-2/PKCα signalling and cross-talk with TGFβ
Source: Cardiovasc Res. 2017 Sep 6;113(13):1639–52. doi: 10.1093/cvr/cvx178 (PMC5852548; doi:10.1093/cvr/cvx178)
Supplement: Supplementary Data [file cvx178_figure_s2.pdf]

A

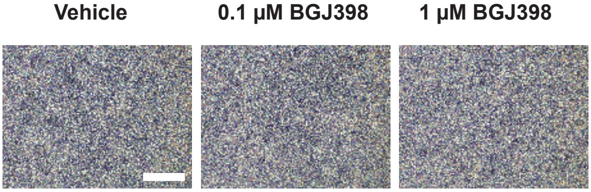

Vehicle
0.1 μM BGJ398
1 μM BGJ398

B

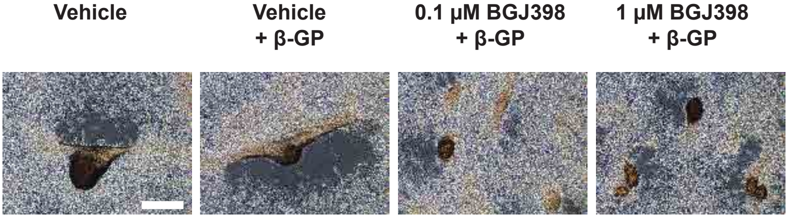

Vehicle
Vehicle
+ β-GP
0.1 μM BGJ398
+ β-GP
1 μM BGJ398
+ β-GP
