# Supplementary material for: Regulation of vascular smooth muscle cell calcification by syndecan-4/FGF-2/PKCα signalling and cross-talk with TGFβ
Source: Cardiovasc Res. 2017 Sep 6;113(13):1639–52. doi: 10.1093/cvr/cvx178 (PMC5852548; doi:10.1093/cvr/cvx178)
Supplement: Supplementary Data [file cvx178_online_supplement_figure_legends.docx]

**Supplemental Figure Legends**

**Figure S1. FGF-2 inhibits VSMC mineralization in a time-dependent manner.** VSMCs were cultured with 5 mM β-GP ± 50 ng/ml FGF-2. FGF-2 was included in the culture medium of β-GP-treated cells continuously, or for specific time-points during the β-GP-treatment protocol. Cells were stained with alizarin red (bar=500 µm) and mineral deposition quantified (*n*=4 independent experiments). Data are means ± SEM. Data were normalized using log_10_ and analyzed using a one-way ANOVA with Dunnett post-hoc tests. *P<0.05compared to β-GP control.

**Figure S2. BGJ398 does not induce matrix mineralization in the absence of raised phosphate levels, nor in non-mineralizing VSMCs.** (**A**) Confluent VSMCs were cultured with BGJ398 (0.1 or 1 μM) in the absence of raised phosphate levels for up to 11 days, which is the length of time needed to detect mineralization in cells cultured with 3 mM β-GP and vehicle (DMSO) or BGJ398 (see *Figure 2C)*. Phase-contrast images of cells stained with alizarin red (bar=500 µm). (**B**) VSMCs that do not mineralize in the presence of raised phosphate levels were cultured with 5 mM β-GP and BGJ398 (0.1 or 1 μM) for 18 days. Cells cultured with vehicle (DMSO) ± 5 mM β-GP were used as controls. Phase-contrast images of cells stained with alizarin red (bar=500 µm).

**Figure S3. Knocking-down syndecan-4 or PKCα expression does not induce matrix mineralization in the absence of raised phosphate levels.** (**A**) VSMCs transfected with control siRNA, syndecan-4 siRNA or PKCα siRNA were cultured in control media for up to 10 days. VSMCs transfected with control siRNA and β-GP were used as controls. Cells were stained with alizarin red (bar=500 µm) and mineral deposition was quantified (*n*=3 independent experiments). Data are means ± SEM. Data were normalized using log_10_ and analyzed using a one-way ANOVA with Dunnett post-hoc tests. *P<0.05. (**B**) A preparation of nodule-forming VSMCs that do not mineralize in the presence of raised phosphate levels were transfected with negative control or syndecan-4 siRNA, and cultured with 5 mM β-GP for 12 days. Cells were stained with alizarin red (bar=500 µm). (**C**) VSMCs were cultured with Gö6976 (1 μM) in the absence of raised phosphate levels for up to 11 days, which is the length of time needed to detect mineral deposition when cells are cultured in the presence of Gö6976 and 3 mM β-GP (as in *Figure 7B)*. Phase-contrast images of cells stained with alizarin red (bar=500 µm). (**D**) VSMCs that do not mineralize in the presence of raised phosphate levels were cultured with 5 mM β-GP and Gö6976 (1 μM) for 18 days. Cells cultured with vehicle (DMSO) ± 5 mM β-GP were used as controls. Phase-contrast images of cells stained with alizarin red (bar=500 µm).

**Figure S4. FGF-2 does not induce syndecan-4 expression in confluent VSMCs.** Confluent VSMCs were serum-starved for 2 hours, and then incubated with 50 ng/ml FGF-2 for 24 hours. Cells incubated with an equivalent volume of vehicle were used as controls. Syndecan-4 mRNA expression was measured using qPCR. Data are mean ± SEM (*n*=3 independent experiments).

**Figure S5. Differential regulation of PGs during VSMC mineralization.** VSMCs (day 0) were incubated ± 3 mM β-GP for up to 14 days. The mRNA levels of glypican-4, biglycan, decorin, versican, osteoglycin, lumican and betaglycan were measured using qPCR. Data are expressed relative to day 0. Data are mean ± SEM (*n*=9 independent experiments). Data were normalized using log_10_ and analyzed using 2-way ANOVA with Sidak post-hoc tests. *P<0.05.
