# Supplementary material for: Regulation of vascular smooth muscle cell calcification by syndecan-4/FGF-2/PKCα signalling and cross-talk with TGFβ
Source: Cardiovasc Res. 2017 Sep 6;113(13):1639–52. doi: 10.1093/cvr/cvx178 (PMC5852548; doi:10.1093/cvr/cvx178)
Supplement: Supplementary Data [file cvx178_revised_online_supplement_methods.docx]

**Online Supplement**

**Methods**

*Immunohistochemistry*

Human coronary arteries were obtained with informed consent from the explanted heart at the time of cardiac transplantation (patient details are provided in *Table S1*). Approval from the Local and National Research Ethics Committee (STH 16346, 12/NW/0036) was granted for human tissue use. This investigation conforms to the principles outlined in the Declaration of Helsinki. Artery segments were fixed for 24 hours in 10% (v/v) buffered formalin before being embedded in wax. Tissue sections were de-plasticized in xylene and hydrated through graded concentrations of ethanol to water. Endogenous peroxidase activity was blocked with 3% (v/v) H_2_O_2_ in phosphate buffered saline (PBS) for 30 minutes, and antigen retrieval was performed with 100 mM citric acid monohydrate (pH 6) at 95°C for 20 minutes. Non-specific binding was then blocked by incubation with 2% (v/v) goat serum in PBS for 1 hour at room temperature. Antibodies against FGF-2 (sc-79, Santa Cruz, USA) or syndecan-4 (#3644, Biovision, USA) were diluted in 2% (v/v) goat serum in PBS to give a final concentration of 0.8 µg/ml and 1.25 µg/ml respectively, and incubated with the tissue at 4°C overnight. Tissue incubated with 1.25 µg/ml non-immune rabbit IgG (Sigma-Aldrich, UK) were used as controls. Following three washes in PBS, the tissue was incubated with an anti-rabbit biotin-conjugated secondary antibody (1:200 dilution in 2% (v/v) goat serum in PBS; Dako, Denmark) and positive immunoreactivity was detected using the ABC system (Dako, Denmark) and 3’3’-diaminobenzidine (Sigma-Aldrich, UK); nuclei were counterstained with Mayer’s hematoxylin (Leica Biosystems, UK). Calcification in consecutive tissue sections was analyzed by von Kossa staining. Images were acquired using a 20x/0.80 Plan Apo objective using the 3D Histech Pannoramic 250 Flash II slide scanner.

*Alizarin red staining*

Mineral deposition by cultured VSMCs was confirmed by staining with 40 mM alizarin red (pH 4.1)^1^. In brief, cells were fixed for 20 minutes in 2% (v/v) formaldehyde with 1% (w/v) sucrose, in PBS. Following a single wash in dH_2_O, cells were stained under agitation with 40 mM alizarin red (pH 4.1) for 20 minutes. Cells were then washed a further 4-6 times in distilled water and allowed to dry overnight. Images were captured using a digital camera (Olympus DP70) attached to an IX51 inverted microscope (Olympus).

To elute the alizarin red stain and quantify mineral deposition, cells were placed on a shaking platform for 30 minutes in 10% (v/v) acetic acid. Cells were collected, and with the acetic acid, heated at 85°C for 10 minutes. Samples were then cooled on ice and centrifuged at 20,000 xg for 15 minutes at 4°C. The supernatant was collected and 10% (v/v) sodium hydroxide was added to each sample to neutralize the acid. Absorbance was read at 405 nM using an MRX II absorbance reader (Dynex Technologies, UK). The absorbance of three separate aliquots of each individual sample was measured and averaged to produce one data-point. The absorbance values were as follows for VSMC mineralization: early mineralization (0.09-0.2), mid mineralization (0.21-0.6) and late mineralization (≥0.61).

*Immunoblotting*

VSMC protein was solubilized in lysis buffer (20 mM Tris-HCl pH 7.6, 150 mM sodium chloride, 1 mM EDTA, 1% (v/v) Igepal, 50 mM sodium fluoride, 1 mM sodium orthovanadate, 1 mM sodium pyrophosphate (all from Sigma-Aldrich (UK)) and 1x protease inhibitor cocktail set 1 (Calbiochem, UK)) and quantified using the Pierce BCA protein assay kit (Thermo Scientific, UK). Samples (15-50 µg) were run on 10% or 15% SDS-PAGE gels and blotted with specific primary antibodies overnight at 4°C (see *Table S2* for antibody details). Membranes were incubated for 1 hour with HRP-conjugated secondary antibodies (1:1000) (Dako, Denmark), washed, and then incubated with Enhanced Chemiluminescent Western Blotting Substrate (Thermo Scientific, UK) or UptiLight US WBlot HRP Chemiluminescent Substrate (Interchim, France) for 5 minutes. Membranes were imaged on the ChemiDoc XRS System (Bio-Rad, UK). Membranes were stripped in 25 mM glycine in 1% (w/v) SDS (pH 2) for 20 minutes at room temperature and re-probed for a second primary antibody where required. All membranes were stripped and re-probed for β-actin to confirm equal protein loading.

*RNA isolation and quantitative polymerase chain reaction (qPCR)*

RNA was isolated from VSMCs using the RNeasy Mini Kit (Qiagen, UK) and any remaining genomic DNA was removed by DNase treatment (Invitrogen™, Life Technologies, UK). RNA was reverse transcribed to generate cDNA using a Taqman® kit (Invitrogen™, Life Technologies, UK) in the following reaction: 1 μg RNA, 1x reverse transcriptase buffer, 5 mM MgCl_2_, 2 mM dNTP mix, 1.67 μM random hexamers, 0.5 U/µl RNase inhibitor, 1.2 U/μl reverse transcriptase. RNA was reversed transcribed using a Mastercycler gradient PCR machine (Eppendorf) and the following heat cycles: 25°C for 10 minutes, 48°C for 30 minutes and 95°C for 5 minutes.

qPCR was performed using 1x SYBR Green PCR master mix (Applied Biosystems, Life Technologies, UK), 0.8 μM of sense primer, 0.8 μM anti-sense primer and 1.5 μl of cDNA per reaction. All primers were designed using Primer3 software (Just Bio) and purchased from Eurogentec S.A (Belgium), with the exception of the peptidylprolyl isomerase A (PPIA) primers which was purchased from Primer Design (UK). Primer sequences are shown in *Table S3*. Samples were processed using a CFX96 or CFX384 Real-Time PCR system (Bio-Rad, UK) and the following heat cycles: 50°C for 2 minutes, 95°C for 10 minutes, 40 cycles of 95°C for 15 seconds, 60°C for 1 minute and 72°C for 15 seconds, followed by 1 cycle of 95°C for 15 seconds, 60°C for 20 seconds and 95°C for 15 seconds. All samples were amplified in duplicates and averaged to produce one data-point. The expression of the tested gene was then normalised to the reference genes (ribosomal protein L12 (RPL12) and PPIA) using the comparative C_t_ method. The expression of RLP12 and PPIA were consistent during the osteogenic differentiation and mineralization of bovine VSMCs.

**References**

1. Collett GD, Sage AP, Kirton JP, Alexander MY, Gilmore AP, Canfield AE. Axl/phosphatidylinositol 3-kinase signaling inhibits mineral deposition by vascular smooth muscle cells. *Circ Res* 2007;**100**:502-509.

**Table S1. Human coronary artery specimen patient details**

| **Patient no.** | **Sex** | **Age** | **Etiology** | **Calcification (Y/N)** |
| --- | --- | --- | --- | --- |
| 120 | Male | 45 | Alcoholic cardiomyopathy | Y |
| 142 | Male | 59 | Ischemic heart disease | Y |
| 161 | Male | 59 | Ischemic heart disease | Y |
| 167 | Male | 46 | Ischemic heart disease | Y |
| 168 | Female | 47 | Ischemic cardiomyopathy | Y |
| 143 | Male | 58 | Ischemic cardiomyopathy | Y |
| 150 | Male | 53 | Idiopathic cardiomyopathy | Y |

**Table S2. Antibodies used for immunoblotting**

| **Protein** | **Blocking agent** | **Primary antibody and catalogue number** | **Dilution and duration** | **Secondary antibody** | **Dilution and duration** |
| --- | --- | --- | --- | --- | --- |
| pAkt  (Ser^473^) | 5% (w/v) milk in TBST | Cell Signaling (#4060) | 1:1000; o/n 4°C | Dako goat anti-rabbit HRP | 1:1000; 1 hour RT |
| Akt | 5% (w/v) milk in TBST | Cell Signaling (#9272) | 1:1000; o/n 4°C | Dako goat anti-rabbit HRP | 1:1000; 1 hour RT |
| pErk1/2 (Thr^202^/Tyr^204^) | 5% (w/v) BSA in TBST | Cell Signaling (#4377) | 1:1000; o/n 4°C | Dako goat anti-rabbit HRP | 1:1000; 1 hour RT |
| Erk1/2 | 5% (w/v) BSA in TBST | Cell Signaling (#4695) | 1:1000; o/n 4°C | Dako goat anti-rabbit HRP | 1:1000; 1 hour RT |
| pPKCα  (Ser^657^/Tyr^658^) | 5% (w/v) milk in TBST | Merck Millipore (07-790) | 1:500; o/n 4°C | Dako goat anti-rabbit HRP | 1:1000; 1 hour RT |
| PKCα | 5% (w/v) BSA in TBST | Cell Signaling (#2056) | 1:500; o/n 4°C | Dako goat anti-rabbit HRP | 1:1000; 1 hour RT |
| pSmad2  (Ser^465/467^) | 5% (w/v) BSA in TBST | Cell Signaling (#3108) | 1:500 or  1:1000; both o/n 4°C | Dako goat anti-rabbit HRP | 1:1000; 1 hour RT |
| Smad2 | 5% (w/v) BSA in TBST | Cell Signaling (#5339) | 1:1000; o/n 4°C | Dako goat anti-rabbit HRP | 1:1000; 1 hour RT |
| Syndecan-4 | 5% (w/v) BSA in TBST | Santa Cruz (5G9),  sc-12766 | 1:200; o/n 4°C | Dako rabbit anti-mouse HRP | 1:1000; 1 hour RT |
| FGF-2 | 5% (w/v) milk in TBST | Santa Cruz (147), sc-79 | 1:200; o/n 4°C | Dako goat anti-rabbit HRP | 1:1000; 1 hour RT |
| β-actin | 5% (w/v) milk in TBST | Sigma-Aldrich (#A1978) | 1:10,000; o/n 4°C | Dako rabbit anti-mouse HRP | 1:1000; 1 hour RT |

o/n, overnight

**Table S3. Primer sequences**

| **Gene family** | **Gene** | **Sense primer (5’-3’)** | **Anti-sense primer (5’-3’)** | **Product size (bp)** |
| --- | --- | --- | --- | --- |
| **PG core proteins** | Biglycan | GCTTCGCAACATGAACTGC | CGAAGGTAGTTGAGCTTCAGG | 99 |
|  | Betaglycan | CACAGATGTTGCCCTGTCC | AGCTCCATCCCAGAGTAGCC | 99 |
|  | Decorin | TGGATTGAACCAGATGATCG | CAATGCGGATGTAGGAGAGC | 110 |
|  | Glypican-4 | TTGCAAGAGATGTGGTGAGC | AACAGGGTTTCACAGTCACG | 120 |
|  | Lumican | AGGTCATCACCAAACTGTGC | GGCACACTCTTCAGTTTCAGC | 92 |
|  | Osteoglycin | CAATGCTTTGGAATCTGTGC | ACTGGTGTCATTAGCCTTGC | 118 |
|  | Syndecan-1 | AGTGTGGCCGTAAACATGC | TGAGGTGATATCTGGCAAAGC | 102 |
|  | Syndecan-2 | CTCCATTGAAGAAGCTTCAGG | AGCTCTGGACTCTCTCCATCC | 99 |
|  | Syndecan-3 | ACAATGCCATCGACTCAGG | CAGGAAGGCAGCAAAGAGG | 125 |
|  | Syndecan-4 | GGTGTCCATGTCTAGCACAGC | GAGGAAGACGGCAAACAGG | 109 |
|  | Versican | ACCTTAATAGCAGCCCATGC | GCATGGTTGAGAAATGACAAGG | 109 |
|  |  |  |  |  |
| **Other** | FGF-2 | TGTGCAAACCGTTACCTTGC | CAACTGGAGTATTTCCTTGACC | 137 |
|  | PKCα | TACGGCGTCCTGTTGTACG | CCATGATGGACTGGAACAGC | 85 |
|  |  |  |  |  |
| **Reference genes** | RPL12 | CAAGGCAACTGGTGATTGG | TTGATGATCAGGGCAGAAGC | 108 |
|  | PPIA | Unknown (purchased from Primer Design, UK) | | Unknown |
